# M2c Macrophages Protect Mice from Adriamycin-Induced Nephropathy by Upregulating CD62L in Tregs

**DOI:** 10.1155/2022/1153300

**Published:** 2022-10-10

**Authors:** Junyu Lu, Shengqiu Lv, Jielong Pang, Tao Qin, Yegui Yang, Weisheng Lu, Zhengzhao Li, Geng Yang, Jianfeng Zhang

**Affiliations:** ^1^Intensive Care Unit, The Second Affiliated Hospital of Guangxi Medical University, Nanning, China; ^2^Department of Emergency Medicine, The Second Affiliated Hospital of Guangxi Medical University, Nanning, China

## Abstract

Regulatory T cells (Tregs) and M2c macrophages have been shown to exert potentially synergistic therapeutic effects in animals with adriamycin-induced nephropathy (AN), a model chronic proteinuric renal disease. M2c macrophages may protect against renal injury by promoting an increase in the number of Tregs in the renal draining lymph nodes of AN mice, but how they do so is unclear. In this study, we used an AN mouse model to analyze how M2c macrophages induce the migration of Tregs. Using flow cytometry, we found that M2c macrophages promoted the migration of Tregs from the peripheral blood to the spleen, thymus, kidney, and renal draining lymph nodes. At the same time, M2c macrophages significantly upregulated chemokine receptors and adhesion molecule in Tregs, including CCR4, CCR5, CCR7, CXCR5, and CD62L. Treating AN mice with monoclonal anti-CD62L antibody inhibited the migration of M2c macrophages and Tregs to the spleen, thymus, kidney, and renal draining lymph nodes. Taken together, our results suggest that M2c macrophages upregulate CD62L in Tregs and thereby promote their migration to inflammatory sites, where they exert renoprotective effects. These insights may aid the development of treatments against chronic kidney disease.

## 1. Introduction

Chronic kidney disease has been recognized as a major public health problem. It presents a worldwide prevalence of 8-16% and a complex pathogenesis, for which effective treatment options are lacking [1–3]. The indicators of chronic kidney disease include albuminuria, urine sediment abnormalities, abnormal renal imaging findings, altered levels of electrolyte or acid-base balance in serum, and glomerular filtration rate slower than 60 mL/minute/1.73 m [4, 5]. Currently, there is still a lack of effective treatments for chronic kidney disease, which causes extremely high mortality when it develops into end-stage renal failure, so there is an urgent need to develop effective treatments.

In recent years, studies have shown that cell-based therapies, including regulatory T cells (Tregs), can slow the progression of kidney disease. The migration of Tregs into the kidney can protect against acute and chronic injury [6, 7]. Moreover, Tregs can protect against autoimmune kidney disease and maintain tolerance to kidney transplantation [8–10]. Studies in mouse models have shown that Tregs induce tolerance to kidney transplantation by suppressing effector T cells and regulating dendritic cell function [9]. Kidney-infiltrating Tregs may help repair ischemic–reperfusion injury by negatively regulating proinflammatory cytokines produced by other T cells [11]. Tregs are a unique type of immunosuppressive cell in the immune system. Tregs are involved in the regulation of most immune responses and have important roles in many physiological processes and diseases, such as immune tolerance, autoimmune diseases, and tumors [12, 13]. A stable population of Tregs that can freely migrate to sites of inflammation or injury is important for managing immune responses [14–18]. Very low Tregs counts are associated with autoimmune or inflammatory diseases, while elevated Tregs numbers may lead to immunosuppression or tumorigenesis [16, 17]. In the noninflammatory state, Tregs are widely distributed in lymphoid and nonlymphoid tissues to prevent the occurrence of abnormal immune or inflammatory activity. In the presence of inflammation, Tregs can express different adhesin and chemokine receptors, allowing them to home to different tissues and sites of inflammation, including secondary lymphoid organs, draining lymph nodes, and certain nonlymphoid tissues such as the skin and gut [14]. The removal of Tregs from noninflamed tissues can avoid pathological immune damage or tumor formation after excessive accumulation of Tregs [19]. Therefore, adoptive therapy of Tregs may be a potential therapy for renal injury.

Previous studies have shown that the development, maintenance, and functional specification of Tregs are regulated by multiple layers of factors, including antigenic and TCR signaling, cytokines, epigenetic modifiers, and transcription factors [20]. In addition, cell-extrinsic factors, such as nutrients, vitamins, and metabolites, as well as cell-intrinsic metabolic programs, also influence Tregs stability, plasticity, and tissue-specific heterogeneity [21]. For example, adenosine can increase the number of Tregs and further promote their immunomodulatory activity [22], and autocrine adenosine signaling can also promote Tregs-mediated renal protection [23]. Interestingly, it was found in a previous study that adoptive transfer of Tregs from healthy mice to kidney-injured mice helped restore the normal architecture of the injured kidney, and Tregs cocultured with M2c macrophages showed upregulation of chemokine receptors, which may be the mechanism by which M2C cells enhanced the migration of Tregs to inflammatory sites [24]. Macrophages are important regulators of tissue homeostasis [25], and the M1/M2 macrophage balance determines the fate of inflamed or injured organs [26, 27]. Alternatively activated macrophages (M2 phenotype), including M2a and M2c subsets, exhibit anti-inflammatory functions *in vitro* and protect against renal injury *in vivo* [28], and M2c macrophages are more potent than M2a macrophages in protecting against renal injury [29].

These findings highlight the need to understand how M2c macrophages regulate Tregs. Here, we explored this regulation in an adriamycin-induced nephropathy (AN) mouse model in an effort to clarify how M2c macrophages protect against kidney damage.

## 2. Methods and Materials

### 2.1. Animals and AN Model

In this study, 36 BALB/c male mice aged 4-6 weeks from the Guangxi Medical University Laboratory Animal Center were acclimated for one week under laboratory conditions of constant temperature (22 ± 2°C) and relative humidity (45 ± 5%). Then, the mice were randomly divided into a control group (12 mice) and an AN group (24 mice). The AN mice were injected with 10.5 mg/kg of adriamycin (Haizheng, Taizhou, China) through the tail vein once, while the control group was injected with an equal volume of saline. The peripheral blood and kidney tissue of mice were collected 2 weeks after adriamycin injection, and each index was detected by enzyme-linked immunosorbent assay (ELISA). These animals were included in the study if the indicators were consistent with manifestations of renal impairment; otherwise, they were excluded from the experiment. All procedures were approved by the Animal Ethics Committee of Guangxi Medical University (Nanning, China).

### 2.2. Cell Culture

Commercial cryopreserved mouse peritoneal M2c macrophages (Bluefbio, Shanghai, China) and mouse thymic Tregs (Bluefbio) were quickly thawed at 37°C in a sterile environment, centrifuged at 225 g for 5 min, and rinsed with medium containing 90% (*v*/*v*) Dulbecco's Modified Eagle Medium- (DMEM-) high glucose and 10% (*v*/*v*) fetal bovine serum with 1% penicillin-streptomycin solution (hereafter “complete DMEM”). Cells were washed twice with fresh medium, resuspended in complete DMEM, and then centrifuged again. Then, cells were resuspended in complete medium and expanded in culture for 2-3 days at 37°C in an incubator with an atmosphere of 5% CO_2_.

### 2.3. Lentiviral Transfection

GFP- and RFP-LC3 lentiviruses (GenePharma, Shanghai, China) were used for lentiviral transfection. We diluted antibodies against mouse CD3e (catalog no. ab16669, Abcam, Cambridge, UK) and mouse CD28 (ab203084; Abcam) in phosphate-buffered saline (PBS) to a final concentration of 5 *μ*g/mL, then coated them onto otherwise untreated 24-well plates (500 *μ*L per well). The coated wells were blocked with 500 *μ*L of 1% bovine serum albumin. Tregs and M2c macrophages were resuspended in complete DMEM to a concentration of 5 × 10^5^ cells/mL, and 2 mL per well (1 × 10^6^ cells/well) was added to the antibody-coated plates. Cells were then placed in an incubator at 37°C for stimulation. After 48 h, activated Tregs and M2c macrophages were collected and resuspended in cell culture medium.

Then, 2-5 × 10^5^ activated Tregs and M2c macrophages were added per well in 24-well plates precoated with anti-mouse CD3e and anti-mouse CD28 antibodies. Cells were gently mixed by pipetting after adding virus (10^6^/mL) at a multiplicity of infection = 10. Plates were centrifuged at 1,000 g for 90 min at room temperature. After centrifugation, plates were placed in an incubator at 37°C for 6 h. Next, we carefully aspirated 350 *μ*L of culture medium (70%), added 1.85 mL of complete cell culture medium up to a final volume of 2 mL, and mixed by pipetting. At 24 h after virus infection, the medium was carefully aspirated, and the virus operation was performed in the same plate. Cells were cultured at 37°C, and fluorescence was observed after 2-3 days using an Olympus IX71 fluorescence inverted microscope. Infection efficiency was measured by flow cytometry on a DXflex flow cytometer (Beckman, Fullerton, CA, USA).

### 2.4. Quantitative Reverse Transcription-Polymerase Chain Reaction (qRT-PCR)

M2c macrophages (1 × 10^6^ cells/mL) and Tregs (1 × 10^6^ cells/mL) in logarithmic growth phase were inoculated into 24-well plates and cocultured for 48 h at 37°C. The expression of chemokine receptors and adhesion molecules (CCR1-11, CXCR3, CXCR5, CX3CR1, CD62L, CD62E, and CD62P) was measured between M2c macrophage+Treg cocultures and Treg monocultures using qRT-PCR.

Total intracellular RNA was extracted using the Trizol extraction kit (Invitrogen), and cDNA was synthesized using the Thermo Scientific RevertAid First Strand cDNA Synthesis Kit (Thermo Fisher) and the primers in Supplementary Table 1. qRT-PCR was performed using a real-time fluorescence quantitative PCR instrument (Applied Biosystems). The 2^-*ΔΔ*CT^ method was used to analyze differences in expression of target genes between M2c macrophage+Treg cocultures and Treg monocultures. The housekeeping gene glyceraldehyde 3-phosphate dehydrogenase (GAPDH) was used as an internal reference.

### 2.5. Enzyme-Linked Immunosorbent Assay

Commercial ELISAs were used to assay, in the serum of AN and control mice, the levels of reactive oxygen species (ROS; Jingmeibio, Jiangsu, China), creatinine (Keshun, Shanghai, China), glutathione (Jianglai, Shanghai, China), urine protein (Jianglai), and blood urea nitrogen (ColorfulGene, Wuhan, China). We prepared standard curves and estimated the corresponding concentration of each analyte according to the optical density of the sample.

### 2.6. Hematoxylin-Eosin Staining

Paraformaldehyde-fixed mouse kidney tissues were embedded in a wax block and sectioned using a Leica DM2235. Tissue sections were immersed in xylene solution for 5 min twice. After hydration through a graded ethanol series, samples were immersed in hematoxylin staining solution. Finally, samples were placed in eosin staining solution for 2 min, and excess staining solution was washed away with 80% ethanol. Samples were dried with a filter paper, and an appropriate amount of neutral gum was quickly added dropwise. The coverslips were then mounted and analyzed on an OLYMPUS BX51 microscope.

### 2.7. Flow Cytometry Detection of Treg Migration in AN Mice

Twelve AN mice were randomly divided into a Tregs treatment group (six mice, each treated with 1.2 × 10^6^ Tregs) and M2c macrophage+Tregs treatment group (six mice, each treated with 1.0 × 10^6^ M2c macrophages + 1.2 × 10^6^ Tregs). After intraperitoneal injection of sodium pentobarbital, mice were sacrificed by cervical dislocation, and the peripheral blood, spleen, thymus, kidney, and renal-draining lymph node tissues were collected, the blood was washed in PBS, and the different organs were cut into small pieces. Blood (0.5-1 mL) was collected from the mouse eyeball into EDTA-coated tubes, and 200 *μ*L of anticoagulated whole blood was added to each sample tube. Erythrocyte lysate was diluted 1 : 10 with distilled water, and 2 mL of diluted lysate was added per 100 *μ*L of whole blood, and lysis of the mixture was allowed to proceed for 8-12 min until the cell suspension became transparent. The suspension was centrifuged at 500 g for 5 min, the cells were washed, the cells were centrifuged again, and the supernatant was removed. The cells were resuspended with 500 *μ*L of PBS, and flow cytometry was performed using a DXflex flow cytometer (Beckman).

### 2.8. Detection of Tregs Migration in AN Mice by Frozen Section

After intraperitoneal injection of sodium pentobarbital, mice were sacrificed by cervical dislocation, and the peripheral blood, spleen, thymus, kidney, and renal-draining lymph node tissues were collected, the blood was washed in buffer, and the different organs were cut into small pieces. Tissues were placed into a sample holder with OCT embedding glue and allowed to stand at 4°C for 5 min to allow the glue to penetrate the tissue. The sample holder containing the tissue blocks was placed in liquid nitrogen for 20 s. The blocks were sectioned in a cryostat into slices 5-10 *μ*m thick. Slices were left standing at room temperature for 30 min, fixed in propionaldehyde for 5 min at 4°C, oven-dried for 20 min, and washed 3 times with PBS. Freezing and mounting were then performed using an aqueous mounting medium (CWBio) containing 4′,6-diamidino-2-phenylindole (DAPI).

### 2.9. Validation of the Regulation of Tregs Migration by M2c Macrophages via CD62L

The expression of CD62L in AN mice was inhibited by DREG-CD62L inhibitor (Invitrogen, Carlsbad, CA, USA). Twelve AN mice were randomly divided into a Tregs treatment group and M2c macrophage+Tregs + DREG treatment group. After intraperitoneal injection of sodium pentobarbital, mice were sacrificed by cervical dislocation, and the peripheral blood, spleen, thymus, kidney, and renal-draining lymph node tissues were collected. The distribution of cell types in the circulation, spleen, thymus, kidney and renal-draining lymph nodes was compared between the two groups of mice using flow cytometry.

### 2.10. Statistical Analyses

Results were expressed as the mean ± standard error, and intergroup differences were assessed for significance using *t* tests. All statistical analyses were performed using GraphPad Prism (GraphPad Software, La Jolla, CA, USA). Differences associated with *P* < 0.05 were considered statistically significant.

## 3. Result

### 3.1. Adriamycin Induced Nephropathy Mouse Model Showed Significant Renal Injury

**Figure 1:**
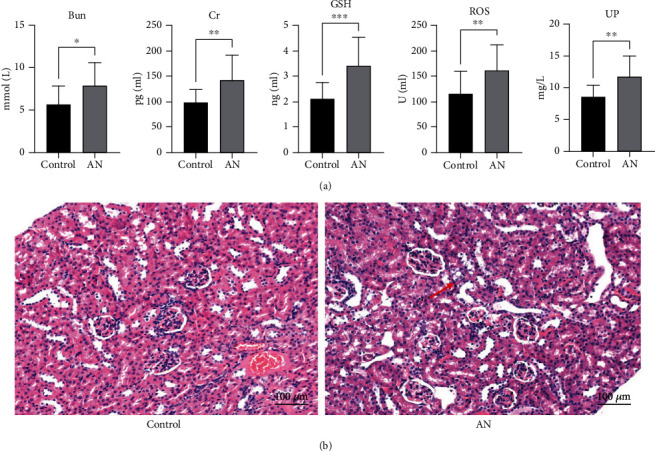
Kidney injury mouse model constructed by adriamycin. (a) The levels of reactive oxygen species (ROS), creatinine (Cr), glutathione (GSH), urine protein (UP), and blood urea nitrogen (BUN) were detected by enzyme-linked immunosorbent assay in adriamycin-induced nephropathy (AN) and control groups (^∗^*P* < 0.05,  ^∗∗^*P* < 0.01, and^∗∗∗^*P* < 0.001). (b) Hematoxylin-eosin staining of mouse kidney tissues in AN and control groups. Red arrows point to damaged cells.

The peripheral blood and kidney tissue of the mice were collected 2 weeks after adriamycin injection, and the indexes were detected by enzyme-linked immunosorbent assay. Compared with healthy mice, AN mice showed higher levels of ROS, creatinine, glutathione, urine protein, and blood urea nitrogen (Figure 1(a)), all typical indicators of renal injury. The renal tissues of AN mice showed obvious renal tubular atrophy and renal interstitial fibrosis, as well as increased levels of inflammation and apoptotic cells (Figure 1(b)). These results indicated that the renal injury mouse model was successfully constructed with adriamycin.

### 3.2. M2c Macrophages Promote Tregs Migration from the Blood to Different Sites

**Figure 2:**
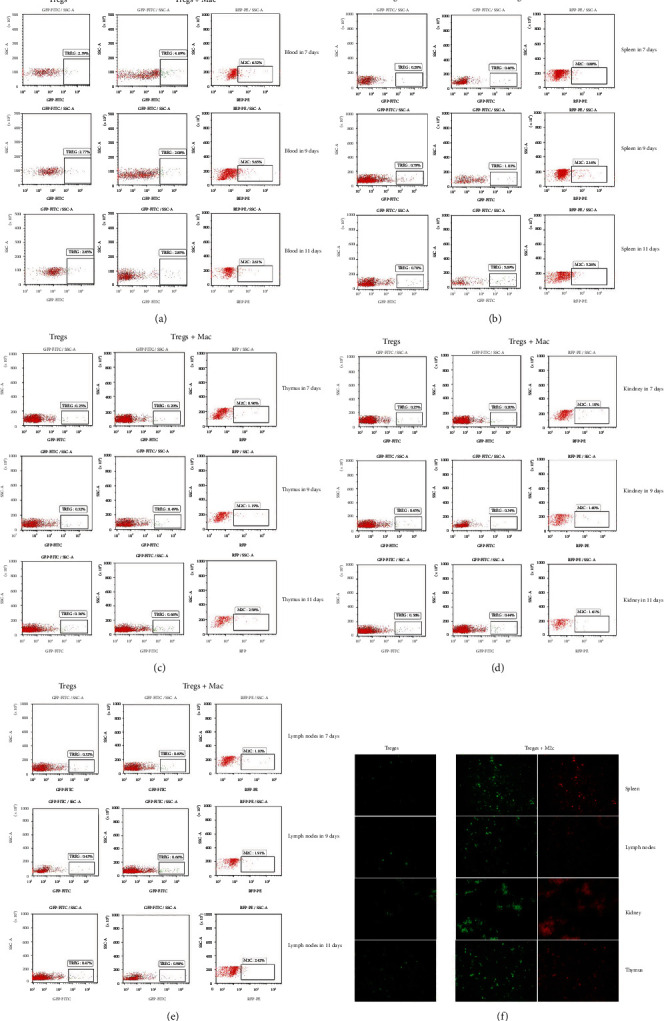
M2c macrophages promote the migration of Tregs from the blood to different sites. (a–e) Flow cytometry of Tregs in the blood, spleen, thymus, kidney, and renal draining lymph nodes in animals treated with Tregs or M2c macrophages+Tregs. (f) Immunofluorescence micrographs of Tregs in the blood, spleen, thymus, kidney, and renal draining lymph nodes in animals treated with Tregs or M2c macrophages+Tregs. FITC: fluorescein; GFP: green fluorescent protein; qRT-PCR: quantitative reverse transcription-polymerase chain reaction; RFP: red fluorescent protein.

We hypothesized that M2c macrophages protect kidneys by regulating the migration of Tregs. We explored this hypothesis in an AN model. Flow cytometry was used to track the migration of Tregs to peripheral blood, spleen, thymus, kidney, and renal-draining lymph node tissue in AN mice that were injected with M2c macrophages+Tregs or Tregs. Numbers of Tregs in peripheral blood decreased gradually over time in animals treated with M2c macrophages+Tregs, and these numbers remained below the numbers in animals treated with either cell type on its own. Conversely, M2c macrophages and Tregs in the spleen, thymus, kidney, and renal-draining lymph nodes gradually increased over time in animals treated with Tregs, and the numbers remained higher than those in animals injected with M2c macrophages+Tregs (Figures 2(a)–2(e)). Immunofluorescence studies confirmed the gradual accumulation of Tregs in the spleen, thymus, kidney, and renal-draining lymph nodes in mice treated with M2c macrophages+Tregs (Figure 2(f)).

### 3.3. M2c Macrophages Promote Tregs Migration via Chemokine Receptors and Adhesion Molecules

To further explore how M2c macrophages regulate Tregs, we cultured mouse peritoneal M2c macrophages and thymic Tregs (Figure S1A) and fluorescently labeled them using recombinant lentivirus (Figure S1B). Then, we cocultured the two cell types and measured the levels of several chemokine receptors that regulate the migration and homing of Tregs.

**Figure 3:**
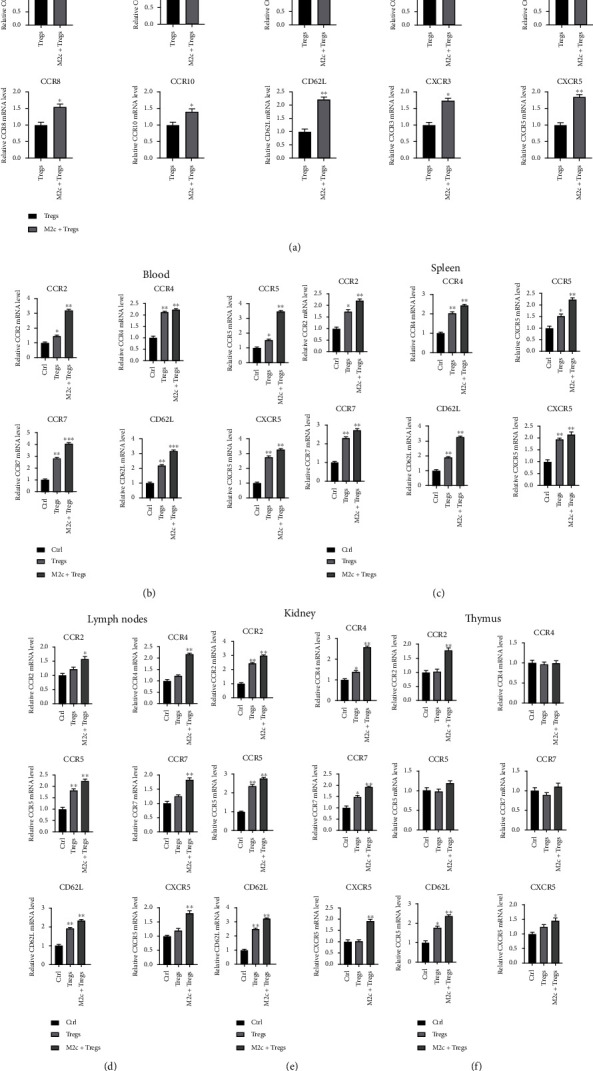
Quantitative reverse transcription-PCR (qRT-PCR) experiments confirmed that M2c macrophages promote the migration of Tregs via chemokines and their receptors. (a) Comparison of chemokine receptor expression between the Tregs-treated group and M2c macrophages+Tregs treatment group, based on qRT-PCR. (b–f) Chemokine receptor expression in the blood, spleen tissue, renal draining lymph nodes, kidney tissue, and thymus tissue from mice of normal control group, Tregs-treated group, and M2c macrophage+Tregs treatment group was compared based on qRT-PCR.

The expression of chemokine receptors varied between Tregs monocultures and M2c macrophage+Tregs cocultures. Among the analyzed chemokine receptors and adhesion molecules, CCR2, CCR3, CCR4, CCR5, CCR7, CCR8, CCR10, CXCR3, CXCR5, and CD62L were significantly upregulated in cocultures, particularly CCR2, CCR4, CCR5, CCR7, CXCR5, and CD62L (Figure 3(a)). M2c macrophages may act through these cytokines to promote the migration of Tregs.

To supplement these in vitro observations, we examined levels of chemokine receptor expression in the blood of AN mice treated with M2c macrophages+Tregs or Tregs. In the peripheral blood and tissue samples, we found that several chemokines, especially CD62L, were upregulated in both the combined treatment group and the Tregs-treated group when compared with controls (Figures 3(b)–3(f) and S2).

### 3.4. M2c Macrophages Promote the Migration of Tregs from Blood to Different Tissues by Upregulating CD62L in Tregs

**Figure 4:**
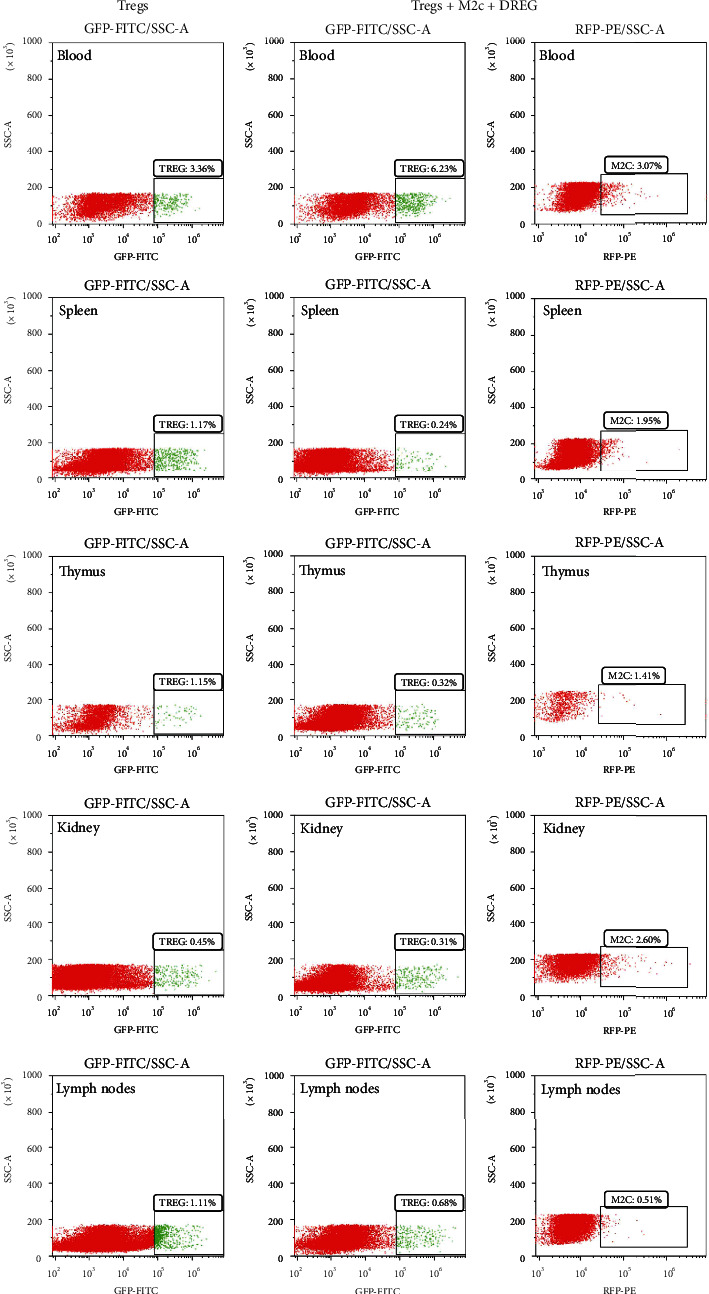
Flow cytometry detection of Tregs in the blood, spleen, thymus, kidney, and renal draining lymph nodes in animals treated with M2c macrophages+Tregs+DREG or only with Tregs. GFP: green fluorescent protein; RFP: red fluorescent protein; FITC: fluorescein isothiocyanate.

To verify whether M2c macrophages upregulate CD62L in Tregs to induce their migration, we examined animals injected with Tregs and animals injected with M2c macrophages+Tregs+DREG. Flow cytometry showed that numbers of Tregs in peripheral blood were higher in animals that received the combined injection than in animals injected only with Tregs. Conversely, numbers of Tregs in the spleen, thymus, kidney, and renal-draining lymph nodes were lower in the animals that received the combined injection than in animals injected only with Tregs (Figure 4), showing that downregulation of CD62L inhibited the migration of Tregs from peripheral blood to spleen, thymus, kidney, and renal drainage lymph nodes.

## 4. Discussion

Although medical technology has made progress in the treatment of kidney injury, effective ways to reverse the disease process are lacking. M2c macrophages and Tregs can protect the kidneys from initial damage and prevent the development of advanced fibrosis in several types of nephropathies, including ischemic kidney injury, diabetic nephropathy, cisplatin-induced kidney injury, AN kidney injury, and lupus glomerulonephritis [30–34]. However, recent studies have come to divergent conclusions about the efficacy and mechanism of M2c macrophages and Tregs in different renal disease models [35–37]. In the present study, we further explored how M2c macrophages protect against kidney injury using the AN mouse model. We first observed the migration of Tregs after treating AN mice with exogenous Tregs alone or in combination with M2c macrophages. We then explored changes in chemokine receptor expression and Tregs migration in AN mice following treatment with Tregs alone or combined with M2c macrophages.

In animal models, Tregs protect against kidney disease, and antibody-mediated depletion of Tregs worsens kidney disease in various models, including acute glomerulonephritis, doxorubicin nephropathy, ischemia-reperfusion injury, and kidney transplantation [38, 39]. Therefore, we speculate that M2c macrophages may protect against AN by regulating the migration of Tregs to injured tissue or organs. When we added exogenous M2c macrophages to mice, we observed that the number of exogenous Tregs in the blood decreased, while the number of Tregs in the spleen, draining lymph nodes, and kidney increased. M2c macrophages promoted the migration of Tregs from the blood into the spleen, thymus, kidney, and renal draining lymph nodes, and this migration was associated with upregulation of chemokine receptors and adhesion molecules such as CCR4, CCR5, CCR7, CXCR5, and CD62L in Tregs.

The role of these chemokine receptors has been reported in many previous studies. Chemokines and their receptors coordinate cell migration and homing in vivo [40, 41]. Locally expressed adhesion molecules and chemokine receptors mediate the migration of Tregs into inflammatory sites [42]. Among them, local injection of a CCR4 antagonist inhibits Tregs migration [43]. CCR5 regulates trafficking and effector functions in memory/effector T lymphocytes, macrophages, and immature dendritic cells [44, 45]. CCR7 can direct lymph node homing of Tregs through high endothelial venules [46, 47], while deficiency in CCR7 causes Tregs to localize abnormally, exacerbating acute nephritis [48]. Moreover, Tregs deficient in CCR7 lose their ability to migrate to lymph nodes and can no longer suppress immune or inflammatory processes [49, 50]. CXCR5-mediated signaling plays a central role in B cell trafficking in lymphoid tissues such as the spleen and lymph nodes [51]. These findings suggest that chemokine receptors promote the migration of Tregs. Additionally, CD62L regulates the ability of Tregs to home to sites of injury or inflammation and to suppress those responses [52]. CD62L and CCR7 also appear to be critical for Tregs migration to inflamed tissues [53]. Therefore, our results suggest that these chemokine receptors and adhesion molecules may help M2c macrophages recruit Tregs to renal tissue to alleviate or block renal injury.

We and others have reported several lines of evidence that CD62L is central to the ability of M2c macrophages to regulate Tregs migration to the kidney. In the present study, treating AN mice with anti-CD62L antibody DREG reduced Tregs migration to the spleen, thymus, kidney, and renal drainage lymph nodes. Consistent with our results, Tregs have been shown to expand more easily in culture and be more responsive to chemokine-driven migration to secondary lymphoid organs when they express CD62L than when they do not [54]. Adoptively transferred Tregs can protect mice against lethal acute graft-versus-host disease and autoimmunity only if they express CD62L [55]. Furthermore, CD62L-expressing Tregs exhibited excellent immunosuppressive effects in different disease models [54–57], and these results suggest the therapeutic potential of upregulating CD62L expression in Tregs for renal injury. In conclusion, our study suggests that M2c macrophages may exert renoprotective effects by upregulating CD62L expression of Tregs and mediating their migration to sites of inflammation, which may contribute to the development of therapeutic approaches for chronic kidney disease. Future studies should explore in detail how M2c macrophages upregulate CD62L in Tregs. Such work may help inform efforts to exploit the therapeutic potential of these macrophages and Tregs against chronic kidney injury, such as through adoptive transfer.

## Data Availability

The data used to support the findings of this study are included within the article and are available from the corresponding authors upon request.

## Supplementary Materials

Supplementary Materials Figure 1: coculture of M2c macrophages and regulatory T cells (Tregs). Figure 2: chemokine expression measured by quantitative reverse transcription PCR (qRT-PCR). Table 1: sequences of primers for quantitative reverse transcription PCR (qRT-PCR).
