# Supplementary material for: M2c Macrophages Protect Mice from Adriamycin-Induced Nephropathy by Upregulating CD62L in Tregs
Source: Mediators Inflamm. 2022 Oct 10;2022:1153300. doi: 10.1155/2022/1153300 (PMC9576407; doi:10.1155/2022/1153300)
Supplement: Supplementary Materials — Figure 1: coculture of M2c macrophages and regulatory T cells (Tregs). Figure 2: chemokine expression measured by quantitative reverse transcription PCR (qRT-PCR). Table 1: sequences of primers for quantitative reverse transcription PCR (qRT-PCR). [file 1153300.f1.docx]

Supplementary Material

# Supplementary Figures

**
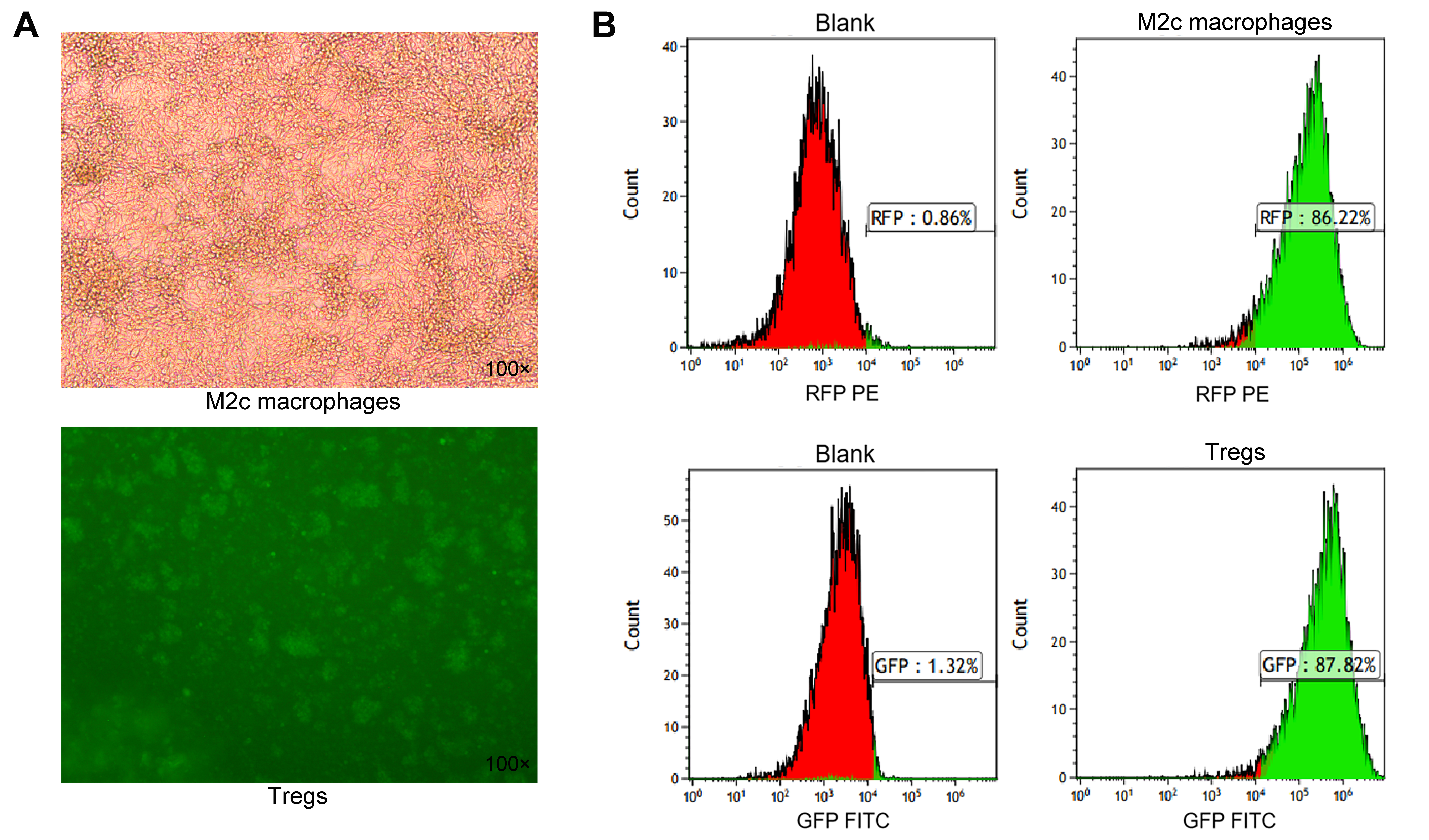
**

**Supplementary Figure 1. Co-culture of M2c macrophages and regulatory T cells (Tregs). (A)** M2c macrophages and Tregs were transfected with lentivirus at 1×10^8^ transducing units/mL. Three days after transfection, M2c macrophages emitted red fluorescence, and Tregs emitted green fluorescence under a fluorescence microscope. **(B)** Efficiency of lentivirus transfection into M2c macrophages and Tregs detected by flow cytometry. The percentage of red fluorescent cells among M2c macrophages transfected with RFP-lentivirus was 86.22%, while the percentage of green fluorescent cells among Tregs transfected with GFP-lentivirus was 87.82%. GFP, green fluorescent protein; FITC, fluorescein; RFP, red fluorescent protein.





**Supplementary Figure 2.** Chemokine expression measured by quantitative reverse transcription PCR (qRT-PCR) in (A) blood, (B) spleen tissue, (C) renal draining lymph node tissue, (D) kidney tissue, and (E) thymus tissue from mice with adriamycin-induced nephropathy. Tregs, regulatory T cells. *P < 0.05, **P < 0.01, ***P < 0.001**.**

## 2 Supplementary Table

**Supplementary Table 1. Sequences of primers for quantitative reverse transcription PCR (qRT-PCR)**

| **Gene** | **Primer sequence** |
| --- | --- |
| GAPDH F | ACCCAGAAGACTGTGGATGG |
| GAPDH R | CCCTGTTGCTGTAGCCAAAT |
| CCR1 F | TTTGGTGTCATCACCAGCAT |
| CCR1 R | GCCTGAAACAGCTTCCACTC |
| CCR2 F | TGGCTGTGTTTGCTTCTGTC |
| CCR2 R | TCTCACTGCCCTATGCCTCT |
| CCR3 F | CTACTCCCACTGCTGCATGA |
| CCR3 R | CTGCTGTGGATGGAGAGACA |
| CCR4 F | GTACTCCAACCTGGGCAAAA |
| CCR4 R | CAGACTGGGTGACAGAGCAA |
| CCR5 F | GGCAAAGACAGAAGCCTCAC |
| CCR5 R | AACCTTCTGCAACACCAACC |
| CCR7 F | GATGCGATGCTCTCTCATCA |
| CCR7 R | TGTAGGGCAGCTGGAAGACT |
| CCR8 F | CAGTATGGCTAACCGCCATT |
| CCR8 R | GCCTTGGTCTTGTTGTGGTT |
| CCR9 F | TTGGCTCTTGACTGTGATGC |
| CCR9 R | GCATGAGAACTGAAGCCACA |
| CCR10 F | GGCTCTCTGAGTGGAAGACG |
| CCR10 R | AAGGCAGCCATTACTGGAGA |
| CCR11 F | GAGGCAGAGCAATGTGACAA |
| CCR11 R | ATTTTCCACGCGTTTGAGTC |
| CXCR3 F | AGCTCTGAGGACTGCACCAT |
| CXCR3 R | CAGTCACTGCTGAGCTGGAG |
| CXCR5 F | CCTCCCAGAACACACTCCAT |
| CXCR5 R | TGCTTGGTCAAGATGACTGC |
| CX3CR1 F | GACGGTTGCATTTAGCCATT |
| CX3CR1 R | TGCTCAGAACACTTCCATGC |
| CD62L F | AAACCCATGAACTGGCAAAG |
| CD62L R | CGCAGTCCTCCTTGTTCTTC |
| CD62E F | AGCCCAGAGCCTTCAGTGTA |
| CD62E R | AACTGGGATTTGCTGTGTCC |
| CD62P F | CACCAATGTGTGAAGCCATC |
| CD62P R | ACATTGCACCCCTGGAGTAG |

Abbreviations: F, forward; R, reverse.
